# Organization of brain networks governed by long-range connections index autistic traits in the general population

**DOI:** 10.1186/1866-1955-5-16

**Published:** 2013-06-27

**Authors:** Pablo Barttfeld, Lucía Amoruso, Joaquín Ais, Sebastián Cukier, Luz Bavassi, Ailin Tomio, Facundo Manes, Agustín Ibanez, Mariano Sigman

**Affiliations:** 1 Physics Department, Laboratory of Integrative Neuroscience, FCEyN UBA and IFIBA, Conicet, Pabellón 1, Ciudad Universitaria, 1428 Buenos Aires, Argentina; 2 Cognitive Neuroimaging Unit, Institut National de la Santé et de la Recherche Médicale (INSERM), 91191 Gif sur Yvette, France; 3 Institute of Cognitive Neurology (INECO), Favaloro University, Buenos Aires, Argentina; 4 Programa Argentino para Niños, Adolescentes y Adultos con Condiciones del Espectro Autista (PANAACEA), Buenos Aires, Argentina; 5 UDP-INECO Foundation Core on Neuroscience (UIFCoN), Diego Portales University, Santiago, Chile; 6 Universidad Torcuato Di Tella, Almirante Juan Saenz Valiente 1010, Buenos Aires C1428BIJ, Argentina

**Keywords:** Autism spectrum disorders, Electroencephalography, Autistic traits, Synchronization likelihood, Small world, Long-range connections

## Abstract

**Background:**

The dimensional approach to autism spectrum disorder (ASD) considers ASD as the extreme of a dimension traversing through the entire population. We explored the potential utility of electroencephalography (EEG) functional connectivity as a biomarker. We hypothesized that individual differences in autistic traits of typical subjects would involve a long-range connectivity diminution within the delta band.

**Methods:**

Resting-state EEG functional connectivity was measured for 74 neurotypical subjects. All participants also provided a questionnaire (Social Responsiveness Scale, SRS) that was completed by an informant who knows the participant in social settings. We conducted multivariate regression between the SRS score and functional connectivity in all EEG frequency bands. We explored modulations of network graph metrics characterizing the optimality of a network using the SRS score.

**Results:**

Our results show a decay in functional connectivity mainly within the delta and theta bands (the lower part of the EEG spectrum) associated with an increasing number of autistic traits. When inspecting the impact of autistic traits on the global organization of the functional network, we found that the optimal properties of the network are inversely related to the number of autistic traits, suggesting that the autistic dimension, throughout the entire population, modulates the efficiency of functional brain networks.

**Conclusions:**

EEG functional connectivity at low frequencies and its associated network properties may be associated with some autistic traits in the general population.

## Background

In the seminal paper in which Kanner first characterized autism, he noticed that some of the symptoms observed in children were shared at sub-threshold levels by their parents [1]. This original observation has been confirmed by several studies [2,3]. Today, the predominant view is that autism spectrum disorder (ASD) is not an all-or-nothing condition; instead, its severity is graded and can be quantified through several diagnostic assessments [4-6]. The graded nature of ASD and the progression of autistic traits in the general population suggest that ASD constitutes the extreme of a dimension traversing through the entire population [7,8]. This has led to the development of behavioural measures seeking to evaluate related traits in the general population, such as the Social Responsiveness Scale (SRS) [7], and to use this dimensional approach to study ASD [7-9].

There is increasing evidence that ASD could be a condition of altered brain connectivity [10-13], including a reduced corpus callosum [14] and diminished long-range functional connectivity [12,15,16], producing a system that is ineffective for integrating information [17-19]. Functional brain networks of ASD compared to typical subjects in the resting state (that is, during free thought) showed qualitatively different organizations at the group level, which broadly reflects a deficit in long-range connectivity, especially along the long-distance fronto-posterior axis [16,18]; see [19] for a review. Most of the knowledge about ASD brain connectivity has been acquired through the use of functional magnetic resonance imaging (fMRI); however, changes in connectivity based on stationary electroencephalography (EEG) measures can also reliably discriminate between ASD and control populations [16]. Compared to fMRI, EEG has remarkable economical and practical advantages [20], such as the much less stressful set-up and a short application time, making it better for large-scale screening, especially with a resting-state paradigm. There are theoretical and practical motivations for using the resting state to assess clinical populations [20,21]. Of these there are the relatively short data-acquisition times, a simple set-up, the subject does not need to be stimulated, behavioural responses do not need to be collected (allowing a broader sampling of patient populations) and a better signal-to-noise ratio compared to task-related protocols [21].

To assist in the diagnosis of ASD, it is essential to find robust brain biomarkers that characterize ASD as the upper extreme of a dimension across the entire population. To date only one piece of research has addressed this issue: it showed that fMRI connectivity in a single link connecting the anterior cingulate cortex and the mid-insula diminishes in strength as the number of autistic traits increases [22]. Here, instead, we study how global brain organization, measured through network properties derived from EEG [23,24], relates to autistic traits in the general population.

In a previous study we showed a diminution of long-range connectivity within the (low-frequency) delta band in an ASD population compared to control groups, leading to a ‘big-world’ organization of brain connectivity in ASD [16]. Here we investigate whether this progression is gradually modulated by the autistic traits of typical subjects by studying resting-state EEG functional networks. Specifically, we hypothesize that: (1) markers for autistic traits in the general population are indexed by the strength of long-range connections (compared to short-range connections), predominantly fronto-occipital connections, (2) changes in connectivity with autistic traits are most prominent in low-frequency bands, which are decoupled in the autistic population [16] and (3) the small-world index significantly decreases with autistic traits in the general population.

## Methods

### Participants and assessment

In this study, there were 74 subjects of similar educational and cultural backgrounds (37 male, 37 female; mean age = 27.33, SD = 5.10; educational level = 19.2 years; SD = 2.94 years). None of the volunteers had a history of neurological or psychiatric conditions as determined by a semi-structured interview (Schedules of Clinical Assessment in Neuropsychiatry) [25]. The interview was conducted by a trained physician in order to exclude any subjects with psychiatric, neurological or sensory impairment, addictions (such as alcohol and drug abuse) or general cognitive impairment. After the subjects were given a complete description of the study, written informed consent was obtained in agreement with the Declaration of Helsinki and the Institution’s ethical committee, which approved this work.

We requested participants to select someone who knew them well, preferably a close relative or partner, to complete the adult version of the SRS questionnaire (SRS-A, [7]). SRS is a 65-item questionnaire completed by an informant who knows the evaluated subject’s preferences and personality. It measures autistic traits across the entire range of severity observed in nature. SRS outputs a score that indexes the severity of social deficits. Higher scores on the SRS indicate greater severity of social impairment. Scores between 60 and 80 are associated with mild forms of autism [7]. Within our population, the scores varied between 11 and 69. Since no tool is currently available in Spanish for the quantitative assessment of autistic impairment across a wide range of severity including the identification of sub-threshold levels of autistic symptomatology, we created an Argentine version of the SRS and ran a pilot study as a first step to full validation. The translation of the adult scale (SRS-A) into Spanish required two simultaneous translations by qualified professionals (author SC and his clinical team at Buenos Aires [26]) with education, training and work experience in the diagnosis of autism spectrum conditions and in the use of psychological tests and assessments in research and clinical settings. After the translation, two back-translations were made and two consensus meetings were held. The final translation was reviewed for clarity by a panel of experts in ASD diagnostics not involved in the translation. After the translation, a pilot study was ran in which the SRS-A test was completed by relatives or close friends of ten adults with an ASD diagnosis, as assessed by the Autistic Diagnostic Observation Schedule (ADOS), and ten typical adults, all with an IQ higher than 85. Interpretation of SRS scores was centred on the total scores. The SRS mean total T-value was highest in the ASD sample (mean SRS = 121.40, SD = 23.71) and lowest in the typically developing sample (mean SRS = 36.9, SD = 9.91; *T*-value = 10.39; *P* = 4.90 × 10^-9^). Also, we observed a significant positive correlation between ADOS and SRS scores within the ASD group (correlation = 0.67, *P* = 0.03, R-squared = 0.42). All ASD subjects scored in the higher range of the mild to moderate group (mild or high functioning autism spectrum conditions) or in the severe range group (autistic disorder or severe cases of pervasive developmental disorder not otherwise specified (PDD-NOS) or Asperger’s syndrome) of the SRS profile sheet. Findings in this pilot study provide adequate support for the application of SRS-A in the assessment of autism traits of the subjects in the present study.

## EEG results

EEG measurements were taken in a Faraday cage with a Biosemi Active Two 128-channel 24-bit resolution system, with active electrodes (the first amplifying stage on the electrode improves the signal-to-noise ratio), digitalized at 512 Hz and low-passed DC-1/5th of the sample rate (-3 dB) by a fifth-order digital sync anti-aliasing filter. There were no additional hardware filters during acquisition. Temporal signals between 5 and 10 minutes were recorded during an eyes-closed rest while subjects sat on a reclining chair in a sound-attenuated room with a dim light. During the experiment, participants and EEG recordings were monitored to ensure that they maintained vigilance and did not fall asleep. After the acquisition, signals were re-referenced to the average of all electrodes. Segments containing movement artefacts were manually deleted (mean length of the remaining time series = 7.90 min, SD = 2.21), followed by an ICA-based rejection of residual artefact-laden ICA-components. After this pre-processing, we filtered the EEG signals on specific frequency bands: delta (0.5 Hz to 4 Hz), theta (4 Hz to 8 Hz), alpha (8 Hz to 12 Hz), sigma (12 Hz to 15 Hz), beta (15 Hz to 25 Hz) and gamma (25 Hz to 35 Hz).

### Data analysis

**Figure 1:**
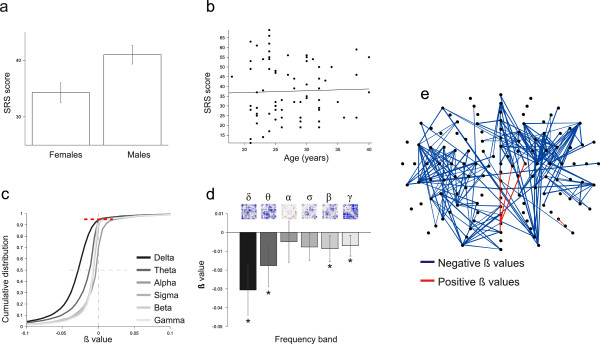
**Autistic traits and functional EEG connectivity.** (**a**) SRS scores. The SRS score is higher for men than women. Error bars show the SEM. (**b**) Scatter plot showing that age does not modulate SRS within our population. (**c**) Cumulative β-value distributions, showing that the entire distributions are shifted to negative values. The dotted red line is a cumulative value of 0.95. (**d**) Mean and SEM of β-value distributions, obtained from independent multivariate regression between SRS score and SL connectivity (including gender and age as regressors of no interest). See Table 1 for mean and standard deviations of the distributions and the *P*-values. All frequency bands except alpha show a bias to negative values. Matrices show thresholded β-values; a blue entry in the matrix is a negative β-value, whereas a red entry is a positive β-value. (**e**) Scalp plot of β-values. A link is traced between two electrodes if that connection is significant (*P* < 0.05, uncorrected) in all frequency bands whose β-values distribution was proved to be significantly shifted to negative values (that is, delta, theta, beta and gamma).

MATLAB (MathWorks Inc, Natick, MA) was used for the analyses. We first explored the relation between SRS score and gender. We calculated the mean SRS score for men and women, and assessed their difference statistically using a t-test for two independent samples. To study the relation between SRS score and age, we conducted both a t-test comparing groups (low SRS score and high SRS score groups, obtained after a median split on the SRS score) and a regression between SRS score and age (Figure 1a, b).

To test whether synchronisation likelihood (SL) connectivity in spontaneous activity between electrodes covaries with SRS score, we conducted a functional connectivity analysis. The synchronisation between all pair-wise combinations of EEG channels was computed for all subjects with the SL method [27]. SL quantifies the probability that a pair of channels is synchronized. For each participant *p* and frequency band *f* we measured a 128 × 128 connectivity matrix *SL*_*f,p*_. A matrix entry *SL*_*f,p*_*(i,j)* indicates the temporal synchronization of the signal measured by electrodes *i* and *j*, for subject *p* at the frequency band *f*, which henceforth is referred to as the functional connectivity. All subsequent analysis and statistics were performed on these *SL*_*f,p *_matrices. To investigate connectivity changes associated with SRS score, we conducted an across-subjects multivariate linear regression, using least squares (as implemented in MATLAB function *regstats()*) between each entry of the matrix *SL*_*f,p *_and the SRS score for each subject, including gender and age of the subject as regressors of no interest. This lead to six matrices of beta (β) values, *B*_*f*_*(i,j),* one per frequency band *f*. For example, a positive value for *B*_*Delta*_*(i,j)* indicates that connectivity between electrode *i* and electrode *j* increases with SRS score for networks measured in the delta band. For visualization we projected all *B*_*f*_*(i,j)* values exceeding a threshold of *P* = 0.05, uncorrected, into a scalp plot (Figure 1e). This threshold is arbitrary: it was used only for visualization and played no role in any statistical analysis.

To assess the *B*_*f *_matrices statistically we performed a bootstrap analysis [28]. We obtained the mean value of each *B*_*f *_matrix, and called it the observed mean *B*_*f *_per frequency band *f*, since this was the β-value obtained experimentally. Then, we calculated the null distribution of β-values for each frequency by band shuffling the SRS scores across participants (thus breaking any possible dependence between functional connectivity and autistic traits across individuals), and repeated the whole regression analysis, to obtain a random mean β-value. We repeated this procedure 5,000 times, obtaining for each frequency band a distribution of 5,000 random mean β-values that approaches a Gaussian distribution. This distribution of random β-values is called a null distribution, or the distribution of expected β-values under the hypothesis of no relation between SRS score and functional connectivity. If any of our observed β-values truly reflects a covariation between functional connectivity and SRS score, its value should be located on the tails of the null distributions. We fitted a Gaussian to each distribution of random β-values to obtain a *Z*-score, by subtracting from the observed β-value the mean value of the fitted Gaussian and dividing it by the standard deviation of the fitted Gaussian. This *Z*-score reflects the distance between the mean of the random distribution of β-values and the observed β-value. We obtained the *P*-values corresponding to the *Z*-scores, and set the *P*-value threshold for significance at 0.05, Bonferroni corrected for multiple comparisons.

To further characterize the β-value distributions, we calculated the standard error of the mean (SEM) for each distribution *B*_*f *_through a jackknife procedure [29], repeating the regression *N* – 1 times (where *N* is the number of subjects), each time excluding a different subject from the analysis. The SEM was then calculated as $\mathrm{std}\left(B_{N}\right)*\sqrt{N-1}$ where *B*_*N *_is the standard deviation of the β-values over the *N* regressions.

To estimate the discriminative power of SL at characterizing autistic traits, we calculated a receiver operating characteristic (ROC) curve [30], categorizing as a ‘hit’ each time a high SRS subject (after a median split) was assigned to the high SRS group, and as a ‘false alarm’ each time a low SRS subject was assigned to the high SRS group. The area under the curve (*A*_ROC_) quantifies how separable the two groups are: *A*_ROC_ = 0.50 means that the two groups completely overlap (along the variable considered), while *A*_ROC_ = 1 indicates that the two groups are perfectly separable by their respective SRS scores.

**Figure 2:**
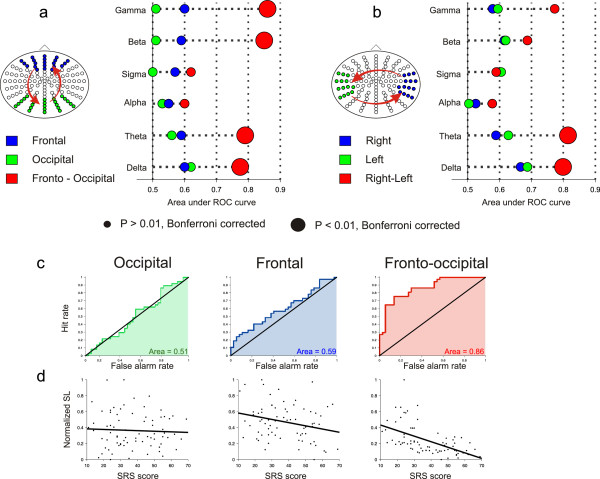
**SL connectivity between and within ROIs.** (**a**) ROC curve value for all frequency bands, between and within frontal and occipital ROIs. Fronto-occipital SL connectivity produced ROC curves showing significant *P*-values for the delta, theta, beta and gamma bands. See Additional file 2: Table S2 for all A_ROC_ values. (**b**) ROC curve value for all frequency bands, between and within right and left ROIs. Left-right SL connectivity produced ROC curves showing significant *P*-values for the delta and theta bands. See Additional file 4: Table S3 for all A_ROC_ values. (**c**) ROC curves after performing a median-split group separation, classifying subjects as high or low SRS score according to their gamma band SL value. Only the fronto-occipital SL shows significant classification (A_ROC fronto-occipital_ = 0.86; A_ROC occipital_ = 0.51; A_ROC frontal_ = 0.59). (**d**) Multivariate regressions obtained by averaging all gamma band SL values within (frontal and occipital) and between (fronto-occipital) ROIs, against SRS score (and gender and age as other regressors). Frontal and occipital SLs do not show significant regression with SRS score (occipital: P = 0.23, R-square = 0.01; frontal: P = 0.31, R-square = 0.001). The fronto-occipital SL, in contrast, does show good regression between SL and SRS score (P < 0.05, R-square = 0.09).

To address the issue of length of connections and their relation with SRS score, we defined four different regions grouping electrodes: frontal, occipital, lateral right and lateral left (Figure 2), covering the contiguous frontal, occipital and temporal electrodes. We then measured connectivity between and within these regions averaging the SL value across electrodes, and we performed a linear multivariate regression (using the least squares method) between SL and SRS score, including age and gender as covariables of no interest. We also conducted a ROC analysis as described above, to explore how well SL values separate between subjects with low and high SRS scores (after grouping subjects into low and high SRS score groups by means of a median split on the SRS score).

### Graph theory metrics

We used graph theory metrics to summarize topological information. The connectivity matrix *SL*_*f,p*_ defines a weighted graph where each electrode corresponds to a node and the weight of each link is determined by the SL of the electrode pair. To calculate network measures, *SL*_*f,p *_matrices were converted to binary undirected matrices by applying a threshold *T*. The arbitrary parameter *T* was chosen so that in all cases the resulting networks had a link density of 0.10, that is, 10% of the total number of possible links in the networks were actually present, to ensure that only the strongest links were present and that the network was not disaggregated into subcomponents [31], and to normalize networks of different subjects by size, in order to avoid spurious effects on the metrics due to network size. After transforming the *SL*_*f,p *_matrix to a binary undirected graph, we calculated the clustering coefficient *C* and the characteristic path length *L* using the Brain Connectivity Toolbox [32]. Combining the metrics *C* and *L*, we calculated the small-world index, *C* / *L*, as an estimate of the small-world properties of the networks. Small world refers to a ubiquitous topological network that has a relatively short (compared to random networks) *L* and high *C*[33]. The small-world index quantifies the optimality of a network in terms of information processing and storage.

To quantify the impact of autistic traits on network properties, we performed two analyses. First we conducted a median-split analysis, grouping subjects as for the SL quantification analysis (conducting a t-test and a ROC analysis). Also we conducted a multivariate linear regression analysis between the small-world index and SRS score (including gender and age as regressors of no interest). To assess the regression analysis statistically, we conducted a bootstrap analysis repeating the methods used to assess the significance of the *B*_*f *_matrices. We obtained the P-value corresponding to the observed small-world index, and set the *P*-value threshold for significance at 0.05, Bonferroni corrected for multiple comparisons.

## Results

First we measured the dependence of SRS score on the demographical covariates age and gender. As expected [4], the women had on average a lower SRS score than the men (women = 34.29 ± 1.66; men = 41.00 ± 1.72; T-value = -1.99; P < 0.05), indicating that men are more likely to have autistic traits than women (Figure 1a). In contrast, age did not show an effect with SRS score, either through a median-split (younger = 38.65 ± 1.99; older = 37.77 ± 1.46; T-value = 0.24; P = 0.80) or through a regression between SRS score and age (β-value = 0.09; P = 0.78) (Figure 1b).

Next we measured the relation between SRS score and connectivity. For each participant in this study, we calculated the synchronization likelihood across all pairs of channels. The element *(i,j)* of the SL matrix provides an estimate of the probability that the time series of electrodes *i* and *j* are related during eyes-closed stationary EEG, which we refer to as functional connectivity. To see SL changes along the autistic dimension we conducted a multivariate regression between each entry of the SL matrix *C(s,p)*_*ij*_ and the values for total SRS, with gender and age as regressors of no interest. A positive β-value β_ij_ indicates that the SL between electrodes *i* and *j* increased as the SRS value increased. Conversely, a negative β-value indicates that SL is greater when the SRS score diminished (Figure 1d).

The regression analysis showed an overall decrease of the mean connectivity (averaged across all electrode pairs) as the SRS score increased. This global decrease in connectivity was observed for all frequency bands, except in the alpha band and was significant for the delta, theta, beta and sigma bands (see Table 1). The effect of the decrease in connectivity with increasing SRS over the entire distribution is clearly depicted by plotting the cumulative value of the entire distribution of β-values (Figure 1d).

**Table 1:** Mean, standard deviation and significance of β-values from the regression between SL and SRS score

**Frequency band**	**Distribution mean and SD**^**a**^	**Significance (bootstrap P)**
Delta	-0.030 ± 0.013	0.01
Theta	-0.017 ± 0.014	0.01
Alpha	-0.005 ± 0.011	0.53
Sigma	-0.007 ± 0.0072	0.10
Beta	-0.008 ± 0.006	0.04
Gamma	-0.007 ± 0.005	0.01

^a^All distributions are shifted towards negative values.

Next, we investigated the hypothesis that long-range connections are more informative about individual autistic traits than short connections. We averaged SL values for all pairs of electrodes within and between frontal, occipital and lateral clusters of electrodes (Figure 2). This electrode clustering broadly defines cortical regions and is not intended (due to the low resolution of the EEG) to define precise boundaries of fine cortical structures. After a median split of subjects according to their SRS score (grouping by high SRS and low SRS scores), we estimated the discriminative power of within and between cluster connectivity to characterize autistic traits, by calculating a ROC curve [30]; we categorize as a ‘hit’ each time a high SRS subject is assigned to the high SRS group, and as a ‘false alarm’ each time a low SRS subject is assigned to the high SRS group. We observed that the SL measured from electrode pairs connecting the frontal and occipital clusters classified subjects better than the SL obtained from electrode pairs within each cluster (Figure 2a). This difference was significant for the SL measured in the delta, theta, beta and gamma bands (P < 0.01, Bonferroni corrected; all A_ROC_ are listed in Additional file 1: Table S1). The SL measured for the alpha and sigma bands, as expected from our previous analysis, did not produce a significant classification of subjects. The negative relation between SRS score and between-clusters SL was also observed from a multivariate regression, using SL as the dependent variable and the SRS score as a regressor (along with age and gender as covariates of no interest). The regression between SRS score and within-clusters connectivity (Figure 2d depicts results for the gamma band; other bands gave similar results) was not significant (occipital: *P* = 0.23, R-square = 0.01; frontal: *P* = 0.31, R-square = 0.001). In contrast, the regression between SRS score and between-regions SL was significant (*P* < 0.05, R-square = 0.09) (Figure 2a), showing that fronto-occipital SL decreases monotonically as the SRS score increases.

The SL between electrode pairs between the left and right clusters also classified subjects better than the SL obtained from electrode pairs within left or right clusters (Figure 2b), although significant differences were only observed in the lower (delta and theta) frequencies (all A_ROC_ values are listed in Additional file 2: Table S2). This suggests that the observed relation between SRS score and between-clusters SL reflects a general effect of distance on SL (see Additional file 3: Figure S1).

**Figure 3:**
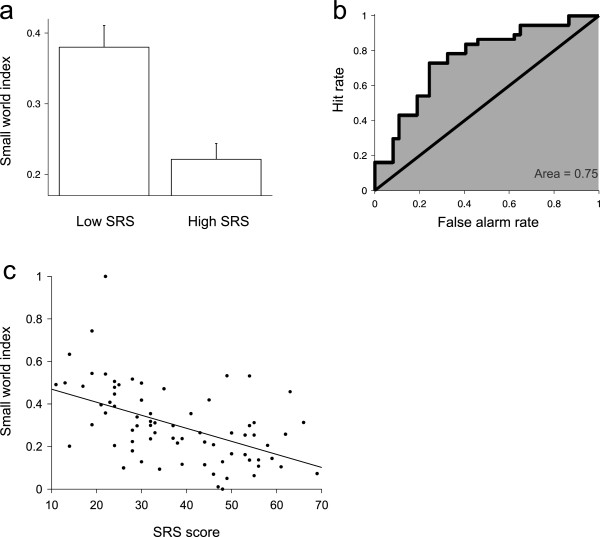
**Topology of networks obtained from delta band *SL***_***f,p ***_**matrices.** (**a**) Small-world indices, after a median-split of subjects into high and low SRS groups. Low SRS subjects have a higher small-world index (low SRS group = 0.38, high SRS group = 0.22; T-value = 4.17; *P* = 0.0001; see Additional file 4: Table S3 for the size effect of all frequency bands). (**b**) ROC curve for the discrimination between low and high SRS score based on the small-world index. A_ROC_ is highly significant (A_ROC_ = 0.74, *P* < 0.001). (**c**) Regression between small-world index and SRS score (and age and gender), showing the negative relation between small-world index and SRS score (β-value = -0.08; *P* = 0.008, R-square = 0.09).

Finally we examined the hypothesis that changes in connectivity result in a different network topology for participants with high or low autistic traits. Specifically we hypothesized that the small worldness of the network, a property which weights the compactness and clustering of a network using optimal architectures for information storage and propagation [17,23], decreased with increasing prevalence of autistic traits. We calculated the small-world index, which quantifies the similarity of the network to a ubiquitous topological network usually referred to as small world [23], and conducted a median-split analysis, grouping subjects as previously, based on their SRS score. High and low SRS score groups showed different mean small-world indices only for the delta band (low SRS group = 0.38, high SRS group = 0.22; T-value = 4.17; *P* = 0.0001; A_ROC_ = 0.75; Figure 3a,b; see Additional file 4: Table S3 for the size effect of all frequency bands). To see whether the differences quantified with the median-split analysis were also observed in a more rigorous analysis not dependent on an explicit group definition along the autistic dimension, we conducted a multivariate regression between the small-world index and SRS score (Figure 3c). For delta band SL matrices, the regression showed a covariation between the small-world index and SRS score (*P* = 0.008, R-square = 0.09), showing that SRS score is negatively related to the optimality of the brain network. Relations between SRS score and the small-world index obtained from all other bands were not significant (see Additional file 5: Table S4 for all effect sizes).

## Discussion

The main purpose of this study was to characterize and compare resting-state functional brain networks in typical subjects along the autistic dimension. In agreement with our three working hypothesis we observed that: (1) the SRS score in the general population is indexed by the strength of connections between EEG electrodes – long-range connections are more predictive than short-range connections of an individual’s SRS score, (2) the lower part of the EEG spectrum is the most informative for individual autistic traits and (3) the small worldness of the network (and hence its optimality for storage and transfer of information) increases as the SRS score diminishes.

Long-range intra-cortical and feedback cortico-cortical connections, which are thought to be altered in ASD, are revealed by the slow cortical potentials of the EEG [34]. Cortico-cortical connections can be roughly classified in two main groups [23,35]: local connections linking neurons in the same cortical area (which are critical in generating functional specificity, that is, information) and long-distance connections between neurons of different cortical regions (which ensure that distant cortical sites can interact rapidly to generate dynamical patterns of temporal correlations, allowing the integration of different sources of information into coherent behavioural and cognitive states) [23,30]. Long-range connectivity provided a good correlate of the individual level of autistic traits, suggesting that functional brain connectivity across distant cortical regions is modulated by the SRS score, diminishing as the SRS score increases and becoming indicative of ASD [16]. The reduced long-range connections may provide a physiological measure for the lack of proper integration of information observed in ASD [36].

Changes in connectivity patterns have an impact on the global organization of a network, which in turn determines the efficiency of information transfer and storage [23,37]. Small-world networks have attracted significant attention during recent decades [33] because they are ubiquitously present in a broad range of natural phenomenon and also because they establish an optimal balance between local specialization and global integration [23]. Our results suggest that the functional networks in the general population are related to the number of individual autistic traits.

In order to become useful tools and assist ASD diagnosis, brain-imaging techniques must find robust biomarkers to characterize ASD and its relation with sub-threshold traits in the general population. Only one piece of research has addressed this issue; Di Martino et al. [22] showed that connectivity within two nodes of the saliency network diminishes in strength as the number of autistic traits increases in neurotypical adults. An estimation of a single connection, however, might not constitute a robust biomarker for characterizing such a complex and diverse condition as ASD, and its performance in actual subject classification remains to be tested. On the other hand, a network approach involving global measures of connectivity and network quality might be better for a robust biomarker [24]. In addition fMRI is not practical for large-scale clinical screening and EEG is a much more suitable, economical and practical tool [20]. Our finding that the optimality of an individual’s EEG network is markedly related to the individual’s SRS score show that it is a good candidate for a biomarker characterizing ASD with practical clinical relevance for large-scale fast screening. Moreover, most brain-imaging evidence suggests that ASD is associated with a diminished connectivity between the frontal lobe and occipitoparietal regions, typically involving default mode network (DMN) nodes such as the ventromedial prefrontal cortex and the precuneus/posterior cingulate [10-13]. It is possible then to have a composite biomarker combining global metrics such as the small-world index and the underlying well-known changes in network topography, a combination that might outperform single-measure biomarkers.

One limitation of the present study is the lack of a measure of specificity regarding possible comorbidities in the autistic traits we measured. The SRS score might capture, along with autistic traits, traits characterizing other psychiatric conditions [38]. Future work should test this network approach through proper classification studies that assess its performance in realistic diagnostic situations and measure its capacity not only to characterize the autistic dimension but to detect it specifically.

## Conclusions

The present study demonstrates that a resting-state EEG can identify robust and monotonic changes associated with SRS score, a graded measure of autistic traits in the general population. Our results show a decrease in functional connectivity, mainly for the delta and theta bands, is associated with an increased number of autistic traits. When inspecting the impact of autistic traits on the global organization of the functional network we found that the optimal properties of the network are inversely related to the number of autistic traits, suggesting that the autistic dimension, throughout the entire population, modulates the efficiency of functional brain networks.

## Abbreviations

ADOS: Autism Diagnostic Observation Schedule; ASD: autism spectrum disorder; EEG: electroencephalography; fMRI: functional magnetic resonance imaging; ROC: receiver operating characteristic; SEM: standard error of the mean; SL: synchronisation likelihood; SRS: Social Responsiveness Scale; ICA: independent component analysis; ROI: region of interest.

## Competing interests

All authors report no financial relationships with commercial interests.

## Authors’ contributions

PB and MS conceived the experiment. PB, SC, LA, JA, LB and AT collected the data. PB and MS analysed the data. PB, SC, MS, AI and FM wrote the paper. All authors read and approved the final manuscript.

## Supplementary Material

Additional file 1: Table S1 A_ROC_ for all frequency bands, for the combination of ROIs frontal, occipital and fronto-occipital. Click here for file

Additional file 2: Table S2 A_ROC_ for all frequency bands, for the combination of ROIs right, left and right-left. Click here for file

Additional file 3: Figure S1 SL connectivity between long and short distances. (a) ROC curve value for all frequency bands, between and within frontal and occipital ROIs. (b) Long-distance SL connectivity produced ROC curves showing significant *P*-values for the delta, theta, beta and gamma bands, while short-distance SL connectivity produced ROC curves showing significant *P*-values only for the delta band. Click here for file

Additional file 4: Table S3 Significance and size effects of the t-test between low and high SRS groups for all frequency bands. Click here for file

Additional file 5: Table S4 Significance and size effects of the regression analysis between the small-world index and SRS score for all frequency bands. Click here for file
