# Supplementary material for: Organization of brain networks governed by long-range connections index autistic traits in the general population
Source: J Neurodev Disord. 2013 Jun 27;5(1):16. doi: 10.1186/1866-1955-5-16 (PMC3698083; doi:10.1186/1866-1955-5-16)
Supplement: Additional file 2: Table S2 — AROC for all frequency bands, for the combination of ROIs right, left and right-left. [file 1866-1955-5-16-S2.doc]

| **Frequency band** | **A_ROC_ fronto-occipital** | **A_ROC_ occipital** | **A_ROC_ frontal** |
| --- | --- | --- | --- |
| Delta | 0,79 | 0,68 | 0,66 |
| Theta | 0,81 | 0,62 | 0,58 |
| Alpha | 0,57 | 0,5 | 0,52 |
| Sigma | 0,58 | 0,6 | 0,59 |
| Beta | 0,68 | 0,61 | 0,61 |
| Gamma | 0,77 | 0,59 | 0,57 |
