# Supplementary material for: Organization of brain networks governed by long-range connections index autistic traits in the general population
Source: J Neurodev Disord. 2013 Jun 27;5(1):16. doi: 10.1186/1866-1955-5-16 (PMC3698083; doi:10.1186/1866-1955-5-16)
Supplement: Additional file 3: Figure S1 — SL connectivity between long and short distances. (a) ROC curve value for all frequency bands, between and within frontal and occipital ROIs. (b) Long-distance SL connectivity produced ROC curves showing significant P-values for the delta, theta, beta and gamma bands, while short-distance SL connectivity produced ROC curves showing significant P-values only for the delta band. [file 1866-1955-5-16-S3.pdf]

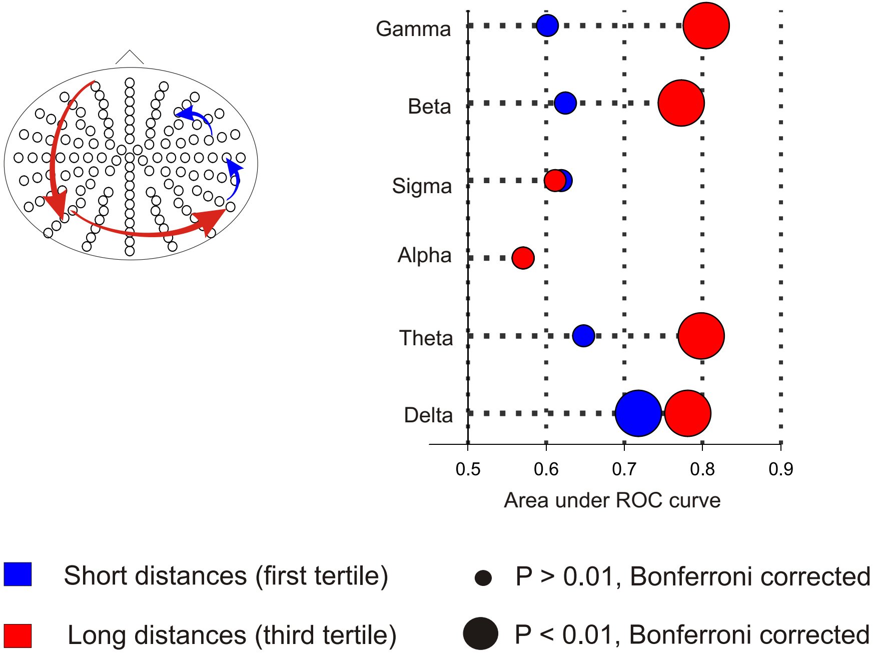

Gamma
Beta
Sigma
Alpha
Theta
Delta
0.5
0.6
0.7
0.8
0.9
Area under ROC curve
Short distances (first tertile)
Long distances (third tertile)
P > 0.01, Bonferroni corrected
P < 0.01, Bonferroni corrected
