# Supplementary material for: Organization of brain networks governed by long-range connections index autistic traits in the general population
Source: J Neurodev Disord. 2013 Jun 27;5(1):16. doi: 10.1186/1866-1955-5-16 (PMC3698083; doi:10.1186/1866-1955-5-16)
Supplement: Additional file 4: Table S3 — Significance and size effects of the t-test between low and high SRS groups for all frequency bands. [file 1866-1955-5-16-S4.doc]

| **Frequency band** | **T-value** | **p-value** |
| --- | --- | --- |
| Delta | 4.17 | 0.0001 |
| Theta | 1.67 | 0.09 |
| Alpha | 0.35 | 0.72 |
| Sigma | -0.1 | 0.91 |
| Beta | 1.31 | 0.19 |
| Gamma | -1.03 | .30 |
