# Supplementary material for: Organization of brain networks governed by long-range connections index autistic traits in the general population
Source: J Neurodev Disord. 2013 Jun 27;5(1):16. doi: 10.1186/1866-1955-5-16 (PMC3698083; doi:10.1186/1866-1955-5-16)
Supplement: Additional file 5: Table S4 — Significance and size effects of the regression analysis between the small-world index and SRS score for all frequency bands. [file 1866-1955-5-16-S5.doc]

| **Frequency band** | **β-value** | **r-square** | **A_ROC_** | **p-value** |
| --- | --- | --- | --- | --- |
| Delta | -0,08 | 0,09 | 0,75 | 8x10^-3^ |
| Theta | -0,02 | 0,02 | 0,6 | 0,2 |
| Alpha | -0,00 | 0,01 | 0,5 | 0,42 |
| Sigma | -0,00 | 0,02 | 0,49 | 0,47 |
| Beta | -0,02 | 0,01 | 0,57 | 0,21 |
| Gamma | 0,01 | 0,02 | 0,42 | 0,36 |
